# Low social capital at work is associated with increased risk of perceived age discrimination: results from a prospective cohort study

**DOI:** 10.1007/s10433-026-00917-w

**Published:** 2026-03-10

**Authors:** Annette Meng, Karen Albertsen, Thomas Clausen, Emil Sundstrup, Lars Louis Andersen

**Affiliations:** 1 National Research Centre for the Working Environment, Copenhagen, Denmark; 2 Team Working Life, Valby, Denmark

**Keywords:** Social psychology, Occupational psychology, Retention of workers, Ageism, Senior workers

## Abstract

Age discrimination at work is negatively associated with older workers’ labour market attachment. High levels of social capital between colleagues (horizontal social capital) and between employees and management (vertical social capital) may prevent age discrimination. *Methods:* We examined the prospective association between social capital at work and perceived age discrimination among 7640 workers aged 50 years or older participating in two waves of the Danish SeniorWorkingLife cohort study. *Results:* Low levels of vertical (risk ratio 3.51 (95% CI: 2.66–4.62)) and horizontal (risk ratio 2.70 (95% CI: 2.04–3.56)) social capital at baseline increased the risk of reporting age discrimination in the labour market at follow-up compared to high levels of social capital. *Conclusion:* The study contributes knowledge that low levels of social capital is associated with increased risk of experiencing age discrimination in the labour market. The results indicate that measures to enhance the social capital in the workplace may help prevent age discrimination and thereby contribute to prolonging working lives of older workers.

## Introduction

The population in many countries is ageing, which reduces the relative size of the workforce and thereby contributes to a lack of qualified labour and potentially posing an economic strain on society. Many Western countries are increasing the state pension age as a measure to overcome this challenge (Eurofound 2021). Nevertheless, despite it being illegal to discriminate against people because of their age in the labour market in the EU (EU 2024), the 2023 Eurobarometer survey showed that 52% of respondents considered age to be a disadvantage in employment representing an increase of five percentage points since 2019 (EU 2023). Examples of age discrimination include: 1) ‘you are being treated unfairly when applying for a job because you are a(n) young/older person’, 2) ‘you have a job where your colleagues treat you badly—such as by calling you names or making jokes at your expense because you are a(n) young/older person’ or 3) ‘your boss refuses you promotion or training because you are a(n) young/older person’ (EU 2024). A Danish study found that 4.8% of a representative sample of workers, aged 50 years or older, reported age-related labour market discrimination (Meng et al. 2022). The estimate is in accordance with the results from a representative study from Norway that found 5–7% of the inhabitants over 50 years in 2020 reporting age-related discrimination of any kind (Oppøyen 2022). Age discrimination in the labour market is not a new phenomenon. Martin and North (2022) offer a perspective on the persistence of ageism by arguing that some egalitarian advocates perceive older individuals as blocking younger people’s access to opportunities and resources (as well as those of other minorities), which can motivate discrimination against older adults despite holding egalitarian values.

Age discrimination has been found to negatively associate with the labour market attachment of older workers (Fasbender & Wang 2016; Meng et al. 2022; Porcellato et al. 2010; Schermuly et al. 2014; Thorsen et al. 2012, 2016). Age discrimination may negatively impact labour market attachment both by posing difficulties for older adults to re-enter the labour market after becoming unemployed and through pushing older workers towards early retirement. Older adults have been found to have longer periods of unemployment (Charni 2021; Wanberg et al. 2016) and lower reemployment rates (Charni 2021; Thomassen et al. 2022; Wanberg et al. 2016). However, overall unemployment among older workers in Denmark has been relatively low in recent years (approximately 2–2.5%). Nevertheless, long-term unemployment becomes more pronounced close to the state pension age: 24.5% of unemployed individuals aged 65–66 are long-term unemployed, compared with approximately 17–20% among those aged 35–64 (Andersen et al. 2025). International literature suggests that the difficulties older adults experience with re-entering the labour market may partly be due to age discrimination in hiring intentions and decisions based on negative attitudes and stereotypes towards older adults (Fasbender & Wang 2016; Kadefors & Hanse 2012; Lu et al. 2011). Age discrimination can also negatively affect the labour market attachment of older workers by pushing them towards earlier retirement (Meng et al. 2022; Schermuly et al. 2014; Thorsen et al. 2012, 2016). Negative attitudes and perceived age discrimination are associated with lower work engagement and job motivation among older workers (Bayl-Smith & Griffin 2014; Furunes & Mykletun 2010; Weber et al. 2019) potentially reducing their motivation to postpone retirement (de Wind et al. 2016). Actively combating age discrimination in the workplace could therefore play a crucial role in extending the labour market participation of older workers.

Workplace-level age discrimination is not an isolated phenomenon but closely linked to the general culture in the workplace such as perceived fairness, helpfulness, and mutual recognition in the process of collaboration. As such, age discrimination may be associated with the workplace level of social capital, and high levels of social capital in the workplace may help prevent age discrimination. In the present study, we therefore examined the prospective association between social capital in the workplace and perceived age discrimination among workers aged 50 years or older.

## Background

Social capital refers to the resources that may arise from the relations within social networks, including workplaces (Almedom 2005; Coleman 1988; Nahapiet & Ghoshal 1998; Putnam 1996). It is expressed through collaboration, helping behaviours, communication, mutual trust, and shared understandings of work tasks (Borg et al. 2014; Nahapiet & Ghoshal 1998). In the literature, a distinction is often made between the social capital in the relations among colleagues and the social capital in the relations between employees or teams and the management (Meng et al. 2018; Pihl et al. 2017; Szreter & Woolcock 2004). Research has confirmed that these represent two distinct components of workplace social capital, referred to as horizontal and vertical social capital, respectively (Oksanen et al. 2010).

Both vertical and horizontal social capital are positively associated with important work-related outcomes such as employee wellbeing (Clausen et al. 2019; Kouvonen et al. 2008; Oksanen et al. 2010), work engagement (Fujita et al. 2016; Meng et al. 2018; Stromgren et al. 2016), job satisfaction (Stromgren et al. 2016), and self-reported job performance (Clausen et al. 2019) which can be expected to positively influence labour market attachment. This is supported by previous research showing that lower levels of work-unit social capital, organisational justice, and quality of management are associated with early retirement (Breinegaard et al. 2017). Cross-sectional studies have found an association between low levels of social capital and undesirable behaviours at the work place such as ‘gossip and slander’ and ‘quarrels and conflicts’ (Kiss et al. 2014), as well as with workplace bullying (Pihl et al. 2017). If these behaviours are directed at older colleagues, they may be perceived as age discrimination as exemplified by the European Commission-statement ‘you have a job where your colleagues treat you badly—such as by calling you names or making jokes at your expense because you are a(n) young/older person’ (EU 2024). Furthermore, a longitudinal study found that incompetence stereotypes towards older workers predicted ineffective work interactions (social capital) between younger/middle-aged and older workers, which again was associated with relational group conflict (Bellotti et al. 2023). Based on these findings, it could be hypothesised that low levels of social capital could lead to age discrimination or behaviours that are interpreted as acts of age discrimination by older workers. Although positive attitudes towards older workers are more common than negative attitudes (Jenkins & Poulston 2014; Jensen & Møberg 2012; Kluge & Krings 2008; Meng et al. 2021, 2022; Van Dalen et al. 2009), particularly negative attitudes regarding the performance of older workers appear to persist (Meng et al. 2021, 2022; Van Dalen & Henkens 2020). Reduced levels of social capital due to incompetence stereotypes towards older workers may thus be a noteworthy challenge in many workplaces.

In addition, perceptions of justice in relationships, and in the process and results related to the distribution of goods (Ambrose & Schminke 2003), have been presented as an important aspect of social capital, particularly in relation to its vertical dimension (Berthelsen et al. 2019). Perception of justice may play an important role in intergenerational conflicts (Hess et al. 2016), and may also be associated with age discrimination or with perceived age discrimination if older workers feel they are treated unfairly, for example, if they are denied promotion or training by their manager and attribute this treatment to their age.

Overall, previous research indicates that low levels of both horizontal and vertical social capital may be associated with perceived age discrimination. However, to date, no research has explicitly investigated the association between workplace social capital and perceived age discrimination. Furthermore, the majority of research on age discrimination of older workers apply cross-sectional studies emphasising the need for research applying longitudinal or prospective designs (Harris et al. 2018).

If low levels of social capital predict age discrimination of older workers, measures to enhance social capital may contribute to eliminating or reducing age discrimination of older workers, and, in turn, potentially support prolonged labour market attachment. Increases in social capital have been found to be associated with a reduction in other undesirable behaviours at the workplace (violence and bullying) (Seppala et al., 2023), and previous research has shown that it is possible to enhance social capital in workplaces (Andersen et al. 2015; Framke et al. 2019; Meng et al. 2020a, b).

The aim of the present study was to examine the prospective association between horizontal and vertical social capital and perceived age discrimination among workers aged 50 years or older. We tested the following two hypotheses:

1. Participants reporting low levels of horizontal social capital at baseline (*T*1) have an increased risk of reporting age discrimination at follow-up (*T*2) compared with participants reporting higher levels of horizontal social capital at baseline (*T*1)
2. Participants reporting low levels of vertical social capital at baseline (*T*1) have an increased risk of reporting age discrimination at follow-up (*T*2) compared with participants reporting higher levels of vertical social capital at baseline (*T*1)

The study thereby contributes with knowledge on factors potentially leading to increased risk of perceived age discrimination in the workplace. Given that it is possible to enhance workplace social capital, and provided that the hypotheses are supported, this study may inform initiatives aimed at reducing or preventing age discrimination at work, and may thereby contribute to prolonging working lives.

## Materials and methods

### Setting

To meet the challenge of labour shortage and strain on the welfare system, Denmark has been and is continuing to increase the state pension age. Denmark is the country in the EU with the steepest raising of the state pension age with an expected state pension age of 70 in 2040 and 74 in 2060 (Euronews 2023). For several years, there has been a tendency towards an increasing proportion of workers aged 65 or older in the workforce. There are indications that it is not only due to the increasing state pension age, but also that more workers want to stay in the labour market for longer (ÆldreSagen 2025). Yet, many workplaces still face severe challenges related to labour shortage and the recruiting of employees (Jensen 2023; Larsen & Jakobsen 2022). This emphasises the need for continued efforts to motivate older workers to postpone retirement.

### Design and participants

We applied a prospective study design using survey data from two waves of the Danish SeniorWorkingLife cohort study (Andersen & Sundstrup 2019). We assessed the prospective association between the level of social capital reported at baseline (*T*1) and perceived age discrimination reported at follow-up (*T*2). The SeniorWorkingLife study is registered in ClinicalTrials.gov (Identifier: NCT03634410) as a cohort study, and the protocol is available as open-access (Andersen & Sundstrup 2019).

At *T*1 in 2020, a total of 18,000 employed individuals aged 50 years or older were drawn as a probability sample by Statistics Denmark and invited to participate with a personal questionnaire link via e-Boks (online digital mailbox linked to the Danish social security number). Participants who replied to the questionnaire in 2020 were invited again in 2022. Data in baseline (*T*1) were collected in the middle of 2020 and follow-up (*T*2) in the middle of 2022. Of the 18,000 invited participants, 12,853 (71%) responded to at least one question in the questionnaire, and 12,488 responded to the question about age discrimination and 12,332 participants responded to the questions on social capital at *T*1. Of these, 1053 participants (7%) reported to have experienced age discrimination at *T*1 and were excluded leaving a total sample of 11,279. Of these, 7640 (68%) participants responded to the follow-up questionnaire two years later (*T*2). Thus, a final sample of 7640 participants was included in the analyses.

The study employs and follows the STROBE reporting guidelines for cohort studies (von Elm et al. 2007).

### Measures

#### Perceived age discrimination

Perceived age discrimination was measured with the question ‘Have you experienced being discriminated against because of your age at the labour market?’ with the following response options: ‘yes’, ‘no’, ‘do not know’, ‘do not wish to reply’. The question was formulated for the purpose of the cohort study and previous research on this question has been published (Meng et al. 2022). The question did not include a specific timeframe for when the participant had experienced age discrimination. Therefore, participants who reported having experienced age discrimination at *T*1 were excluded from the analyses to assess new cases of age discrimination between *T*1 and *T*2. Furthermore, participants who had responded ‘do not know’ or ‘do not want to reply’ were excluded from the dataset as they comprised of less than 2% of the responses. These response categories were invisible to the respondents and only became visible if they attempted to progress in the questionnaire without responding to the question.

#### Social capital

Horizontal social capital was measured with the following three questions adapted from the Danish working environment and health questionnaire (AH 2018; Andersen et al. 2022): ‘How often do you and your colleagues acknowledge each other when working?’, ‘How often do you and your colleagues help each other to achieve the best possible result?’, and ‘How often do you and your colleagues cooperate, when problems arise that need to be solved?’(Cronbach’s α 0.83).

Vertical social capital was measured with the following four questions adapted from the Danish working environment and health questionnaire (AH 2018; Andersen et al. 2022; Sørensen et al. 2025): ‘How often are all employees who are affected by a decision heard?’, ‘How often are all employees treated fairly at the workplace?’, ‘How often is your work acknowledged and appreciated by the management?’, and ‘How often do you receive the help and support, you need, from your immediate manager?’ (Cronbach’s α 0.83).

Pearson’s correlation between the two social capital scales was 0.55, indicating that they can be regarded as related but separate components of social capital.

For all items, the participants were required to respond on a five-point Likert-type scale from ‘always’, ‘often’, ‘sometimes’, ‘rarely’, and ‘never’, which were coded as 100, 75, 50, 25, and 0, respectively. Items on horizontal and vertical social capital were divided into two separate scales where we computed average scores ranging from 0 to 100 with a score of 100 representing the highest level of horizontal and vertical social capital, respectively. Based on the response categories, we categorised both horizontal and vertical social capital into ‘low’, ‘moderate’, and ‘high’ levels. Since the responses to the items on social capital were skewed towards the more positive response options, we categorised scores between 0 and 50 as ‘low’ levels of social capital; scores between 51 and 75 as ‘medium’, and scores between 76 and 100 as ‘high’ levels of social capital. This categorisation can be interpreted with reference to the response options and implies that respondents in the ‘low’ category on average responded ‘Sometimes’ or less frequently to the items on social capital, while respondents in the ‘medium’-category on average responded between ‘Sometimes’ and ‘Often’ to the items on social capital. Finally, respondents in the ‘high’-category on average responded between ‘Often’ and ‘Always’ to the items on social capital. Previous studies have also deployed social capital as a categorical independent variable (Kouvonen et al. 2008; Oksanen et al. 2010).

#### Control variables

In the analyses, we controlled for age and gender, job function, and education. Previous research has found differences between job groups and men and women regarding which attitudes towards older workers they perceive their management to have (Meng et al. 2021). Thus, there could also be differences in perceived age discrimination.

Age, gender and highest completed education were drawn from a national register. Education was categorised into the following three categories based on the register variable *‘highest completed education’* by Statistics Denmark (DST 2024) 1. elementary or high school, 2. vocational education, 3. higher-level education.

Job function was measured with the question ‘what do you work with first and foremost in your daily work?’ in the questionnaire with the following five response options 1. office work, administration, analysis, IT, 2. working with people, service, care, 3. working with processing, producing or moving things, 4. Other, 5. do not know (Andersen & Sundstrup 2019).

#### Statistical analyses

Using the GENMOD procedure of SAS version 9.4, we conducted robust Poisson regression analysis (Chen et al. 2018) to assess the prospective association between social capital at T1 and perceived age discrimination at follow-up. Analyses were stepwise adjusted for the potential confounders in the following statistical models: Model 1 was adjusted for the demographic confounders age and gender and Model 2 was further adjusted for the work-related confounders job function and education. Results are reported as risk ratios (RR) with 95% confidence intervals (CI).

## Results

As shown in Table 1, 55 per cent of the participants were males, and they had a mean age of 58 years. Forty-nine per cent were doing office work, 23 per cent were working with people, 13 per cent were working in the field of production, and 14 per cent had other unspecified job functions. Regarding highest completed education, 19 per cent had an elementary or high school level education, 43 per cent had a vocational education and 38 per cent had a higher-level education.

**Table 1:** Descriptive statistics of the participants

Gender (%)	
Female	44.8
Male	55.2
Age (mean (SD))	58 (4.6)
*Job function (%)*	
Office work	48.9
Working with people	23.4
Working in the field of production	13.2
Other	14.4
*Education (%)*	
Elementary, high school	18.9
Vocational	42.9
Higher-level	38.2
*Horizontal social capital (%)*	
Low (0–50)	7.2
Moderate (51–75)	43.3
High (76–100)	49.6
*Vertical social capital (%)*	
Low (0–50)	25.5
Moderate (51–75)	48.1
High (76–100)	26.4
*Age discrimination (%) (T2)*	
Yes	6.0

n = 7640

Regarding horizontal social capital, the largest proportion of participants reported high levels followed by moderate levels, while only seven per cent reported low levels of horizontal social capital. Regarding vertical social capital, around a fourth of the participants reported low as well as high levels of social capital, while about half of the participants reported moderate levels of vertical social capital (see Table 1). Lastly, six per cent of the participants reported age discrimination at the labour market at *T*2 (see Table 1).

When comparing gender and age subgroups, there were no significant difference between the groups in the proportion reporting age discrimination. Results regarding the subgroups related to work show that a larger proportion of participants doing office work and those working with people, reported age discrimination than participants working in the field of production or had unspecified job functions. Chi-square test showed that there are significant differences between the groups. Furthermore, the results show that a larger proportion of participants with higher level of education reported age discrimination than participants with vocational or elementary/high school level education. Again, Chi-square test showed significant differences between the groups (see Table 2).

**Table 2:** Proportion of participants reporting age discrimination at *T*2 divided by subgroups and associated *p* values from Chi-square tests (*n* = 7640)

	Experienced age discrimination (*T*2) (%)	*P*
*Gender*		
Male (*n* = 4219)	5.8	
Female (*n* = 3421)	6.2	0.48
*Age*		
50–54 (*n* = 1801)	5.2	
55–59 (*n* = 2776)	5.9	
60–64 (*n* = 2353)	6.5	
65 or older (*n* = 710)	6.2	0.47
*Job function*		
Office work (*n* = 3743)	6.7	
Working with people (*n* = 1786)	6.2	
Working in the field of production (*n* = 1008)	5.0	
Other (1103)	3.9	< 0.01
*Education*		
Elementary, high school (*n* = 1344)	5.4	
Vocational (*n* = 3254)	4.9	
Higher-level (*n* = 3042)	7.4	< 0.001
*Horizontal social capital*		
Low (0–50) (*n* = 547)	11.3	
Moderate (51–75) (*n* = 3305)	6.8	
High (76–100) (*n* = 3788)	4.5	< 0.001
*Vertical social capital*		
Low (0–50) (*n* = 1950)	10.4	
Moderate (51–75) (3671)	5.2	
High (76–100) (2019)	3.2	< 0.001

Comparing the social capital groups, results showed that, for both horizontal and vertical social capital, a larger proportion of participants reporting low levels of social capital at *T*1 reported age discrimination in the labour market at *T*2, than those reporting moderate levels of social capital. While those reporting high levels of social capital had the smallest proportion of participants reporting age discrimination at *T*2. Chi-square test showed significant differences between the groups (see Table 2).

When calculating the risk ratios, the results showed that participants who reported low levels of *horizontal* social capital at *T*1 had 2.6 times increased risk of reporting age discrimination at *T*2 than those reporting high levels of social capital, and 1.7 times increased risk compared to those reporting moderate levels of horizontal social capital. Those reporting moderate levels of horizontal social capital had 1.5 times higher risk than those reporting high levels. All risk ratios were statistically significant (see Model 1 in Table 3).

**Table 3:** Risk ratios of reporting age discrimination in the labour market at *T*2 comparing participants with different levels of horizontal and vertical social capital

	Model 1 mean estimate (confidence limits)	Model 2 mean estimate (confidence limits)
*Horizontal SC*		
Low versus high	2.64 (2.01–3.47)***	2.70 (2.04–3.56)***
Moderate versus high	1.54 (1.27–1.86)***	1.50 (1.24–1.82)***
Low versus moderate	1.72 (1.32–2.24) ***	1.80 (1.38–2.35) ***
*Vertical SC*		
Low versus high	3.42 (2.60–4.50)***	3.51 (2.66–4.62)***
Moderate versus high	1.69 (1.28–2.23)***	1.66 (1.26–2.19)***
Low versus moderate	2.02 (1.67–2.45)***	2.11 (1.75–2.56)***

*n* = 7640. Model 1 adjusted for age and gender, Model 2 further adjusted for job function and education, *P < 0.05, **P < 0.01, ***P < 0.001

The risk ratios for *vertical* social capital showed the same pattern of results. Low level of vertical social capital at *T*1 was associated with a 3.4 times increased risk of reporting age discrimination at *T*2 compared to those reporting high levels, and 2.0 times increased risk compared to those reporting moderate levels. Those reporting moderate levels of vertical social capital showed a 1.7 times increased risk of reporting age discrimination at *T*2 compared to those reporting high levels. All risk ratios were statistically significant (see Model 1 in Table 3).

When we further adjusted for job function and education, the risk estimates did not change notably and the significance levels remained the same for both horizontal and vertical social capital (see Model 2 in Table 3).

## Discussion

The aim of the study was to examine the prospective association between horizontal and vertical social capital and perceived age discrimination among workers aged 50 years or older. We tested the following two hypotheses: (1) Participants reporting low levels of horizontal social capital at baseline (*T*1) have an increased risk of reporting age discrimination at follow-up (*T*2) compared with participants reporting higher levels at baseline (*T*1), and (2) participants reporting low levels of vertical social capital at baseline (*T*1) have an increased risk of reporting age discrimination at follow-up (*T*2) compared with participants reporting higher levels at baseline (*T*1).

The results confirm our hypotheses by showing that low levels of both horizontal and vertical social capital at baseline were associated with an increased risk of reporting age discrimination at follow-up.

On average six per cent of the participants reported age discrimination at the labour market at follow-up, with the proportion being up to 11 per cent among those reporting low levels of workplace social capital. Although this implies that approximately nine out of ten older workers in Denmark do not report to have experienced age discrimination at the labour market, on a national scale, this accounts for a substantial number of workers. Furthermore, in the first wave of the SeniorWorkingLife study in 2018, 4.8 per cent of the employed participants reported age discrimination (Meng et al. 2022), indicating that perceived age discrimination is a persistent and slightly increasing problem. Considering the negative association between age discrimination and labour market attachment (Fasbender & Wang 2016; Meng et al. 2022; Porcellato et al. 2010; Schermuly et al. 2014; Thorsen et al. 2012, 2016), these findings highlight the importance of addressing the problem and developing measures to eliminate or reduce age discrimination at the labour market.

Interestingly, results showed that a larger proportion of participants with office-based work reported age discrimination than those working with people or in production. In addition, age discrimination was reported more frequently among participants with higher levels of education than among those with lower levels of education. An earlier study, also drawing on data from the SeniorWorkingLife study, explored which attitudes, participants believed their manager held towards older workers. The study found, that participants with mainly physical work were less likely to report both negative and positive attitudes compared to participants with mainly seated work (Meng et al. 2021). The authors conclude that differences appeared to exist between occupational groups but they called for more research to clarify these differences. Our results supplement these findings by indicating that workers in occupations with mainly seated work may perceive age discrimination to a larger extent. Future research is encouraged to further explore the dynamics of age attitudes and discrimination in different occupations.

Testing the hypotheses, the results showed that low levels of horizontal social capital at baseline were associated with an increased risk of reporting age discrimination at follow-up. This association did not become weaker when controlling for the work-related confounders (job function and education) even though these were significantly associated with perceived age discrimination. The results thus supported Hypothesis 1. Previous cross-sectional studies have found that low levels of social capital are associated with ‘gossip and slander’ and ‘quarrels and conflicts’ at the workplace (Kiss et al. 2014), and workplace bullying (Pihl et al. 2017). If such behaviours are directed at older colleagues, they may be perceived as age discrimination. Our findings elaborate previous findings by showing a prospective association between low levels of social capital and undesirable behaviours at the workplace, more specifically, perceived age discrimination. Our findings also indicate that ensuring high levels of social capital among colleagues may indeed contribute to preventing age discrimination.

In addition, a bidirectional relationship between social capital and age discrimination could be hypothesised, where experiences or perceptions of age discrimination may also have detrimental effects on workplace social capital. This may create a vicious circle emphasising the need to address the issue. The findings by Bellotti et al. (2023) that incompetence stereotypes about older workers are associated with lower levels of social capital in relationships between younger/middle-aged and older workers provides further indications of a potential vicious circle.

Our results also supported Hypothesis 2 by showing that low levels of vertical social capital at baseline were associated with increased risk of reporting age discrimination at follow-up. This association did not diminish when controlling for the work-related confounders (job function and education) even though these were significantly associated with perceived age discrimination. The items we used for measuring vertical social capital emphasised the experience of justice in the relationship between management and employees. As age discrimination can be interpreted as a form of experienced interpersonal injustice, this supports the conclusion, that lower levels of social capital can increase the risk of age discrimination, particularly when low social capital characterises vertical workplace relations. The results therefore seem to support that retention of older workers may be influenced by social capital at the workplace, and particularly by the perception of justice.

As well as showing a prospective association between vertical social capital and age discrimination, our results also showed that a larger proportion of participants reported low levels of vertical than horizontal social capital. This could indicate that low levels of social capital in the relationship with the management are a more widespread problem than low levels of social capital among colleagues. These findings elaborate on previous research showing associations between leadership style and bullying (Skogstad et al. 2011) as well as acts of incivility (Baig & Zaid 2020; Harold & Holtz 2014; Kaiser 2017). They suggest that improving the quality of relationships between management and employees may be particularly important when aiming to prevent age discrimination. This is in line with suggestions made by Albertsen et al. (2024), that the success of a workplace senior policy may depend on the level of workplace social capital and may benefit from transparency and clear connection between intentions, practices, and distributional justice in the award criteria.

Our results reveal that low levels of social capital constitute a risk factor for perceived age discrimination among workers aged 50 years or older. A recent review found only limited evidence for the effect of interventions that aimed to promote more positive attitudes towards older workers; when effects were observed, they were typically confined to explicit attitudes (Sinclair et al. 2024), which are more prone to social desirability bias. Our findings suggest that addressing risk factors such as low levels of social capital may present an alternative approach to reducing age discrimination.

## Practical implications

Previous research has shown that it is possible to increase the social capital at the workplace (Andersen et al. 2015; Framke et al. 2019; Meng et al. 2020a, b). Our results indicate that low levels of vertical social capital are a more widespread problem than low levels of horizontal social capital, indicating that it may be more effective to address the relationship between employees and management. Furthermore, although interventions aimed at increasing both horizontal and vertical workplace social capital may benefit employees generally, it is important to note that perceptions of social capital can vary within teams. Such intra-team disagreement may indicate the presence of subgroups (Meng et al. 2018), who do not feel fully included and who may therefore not benefit to the same extent from increases in social capital. It is therefore important to ensure that the older workers are included in the social networks (Jokisaari et al. 2024). Interventions to enhance the social capital at the workplace, with the aim to prevent age discrimination, may thus benefit from a combination of general measures to strengthen social capital and measures with a more explicit focus on diversity to ensure inclusion of older workers. This could involve leadership training programmes focused on age-inclusive practices, regular feedback sessions between managers and older workers, and mentoring programmes that pair senior employees with younger managers. Additionally, organisations might consider implementing age diversity training for all employees to foster a more inclusive workplace culture and strengthen horizontal social capital.

Finally, the long-term implications of our findings extend beyond the immediate workplace environment. By addressing age discrimination through the enhancement of social capital, particularly vertical social capital, organisations may not only improve the work experiences of older employees but also contribute to the work environment for employees of all other ages along with broader societal goals. Retaining older workers in the labour market could help alleviate pressures on pension systems, transfer valuable knowledge and skills to younger generations, and promote a more age-diverse and inclusive society. Future research could explore these potential long-term effects, examining how improvements in workplace social capital might influence retirement decisions, knowledge transfer, and societal attitudes towards ageing over time.

## Strengths and limitations of the study

A strength of the study is the prospective study design combined with the exclusion of participants reporting age discrimination at *T*1, allowing for conclusions on the direction of the associations found. Furthermore, the prospective study design also reduced the risk of common method bias, where the strength of associations is inflated (Podsakoff et al. 2003), posing a challenge in research relying on self-reports, particularly when the data are collected at one point in time.

Although social capital is often measured at the individual level (e.g. Kouvonen et al. 2008; Kowalski et al. 2010a, b; Ommen et al. 2009; Stromgren et al. 2016), other researchers have questioned the appropriateness of this arguing that social capital should not be considered a property of individuals but rather regarded as a group phenomenon (e.g. Almedom 2005; McKenzie et al. 2002). In our study, we measured social capital at the individual level, which may be regarded as a limitation of the study. However, because we used data from a cohort study, it was not possible to aggregate data at the team or organisational level. Future studies are encouraged to apply multi-level analyses to further explore the association between workplace social capital and age discrimination. This approach would also further reduce the risk of common method bias.

Another strength of the study is the large representative sample of workers aged 50 years or older making it possible to generalise the results to the Danish working population within this age span. Nevertheless, the findings may not generalise to other national contexts and thus need to be scientifically tested and confirmed in those contexts. In addition, the findings may not be applicable to younger workers experiencing age discrimination, and further research is warranted to examine this issue.

Older workers represent a diverse group and previous research has found difference between subgroups of older workers with regard to the experience of age discrimination or stereotypes (Bobbitt-Zeher 2011; DeArmond et al. 2006; Frøyland & Terjesen 2020; Meng et al. 2021; Posthuma & Campion 2009), and which factors associate with retirement (Eyjolfsdottir et al. 2025; Meng et al. 2020a, b). Our sample was not large enough to examine differences between subgroups in the associations between social capital and perceived age discrimination. Future research is encouraged to explore differences between subgroups based on, for example, gender, age, and educational level.

A limitation of the study is that we measured perceived age discrimination, which may not always reflect actual age discrimination. Nevertheless, it can be argued that perceived age discrimination even if it presents a misinterpretation and does not reflect actual discriminatory acts, is associated with negative feelings and therefore likely to affect older workers’ motivation to stay at the workplace and consequently, their attachment to the labour market. This is supported by earlier findings from the SeniorWorkingLife study showing a prospective negative association between perceived age discrimination and labour market attachment, using the same survey question (Meng et al. 2022). Nevertheless, the measure of age discrimination could be improved by including questions on the extent, nature, context, and severity as well as providing a timeframe for the experienced discrimination (e.g. within the past three months). This would allow for more nuanced analyses of the data. Furthermore, future research measuring actual acts of age discrimination would strengthen the conclusions of this study; however, because of ethical and social desirability issues, it is difficult to study actual acts of age discrimination in research.

Finally, we have coded the two independent variables on social capital into three categories. This may be a limitation of the study as we lose information on the two independent variables by doing so. However, by coding the two scales on social capital into categorical variables, we enhance the interpretability of the results which again supports the practical application of the results—both in practical contexts in workplaces and in relevant political contexts. Moreover, previous studies have also operationalised social capital as categorical variables (Kouvonen et al. 2008; Oksanen et al. 2010), which also allows for detection of potential nonlinear associations between social capital and age discrimination.

## Conclusion

The results showed that low levels of both horizontal and vertical social capital at baseline were associated with an increased risk of reporting age discrimination in the labour market at follow-up in a representative sample of Danish workers aged 50 years or older. The study thereby contributes knowledge on factors associated with increased risk of experiencing age discrimination in the labour market. Overall, the results indicate that measures to enhance the social capital at the workplace may help prevent age discrimination and thereby contribute to prolonged working lives of older workers.

## Data Availability

The authors encourage collaboration and use of the data by other researchers. Data are stored on the server of Statistics Denmark, and researchers interested in using the data for scientific purposes should contact the project leader Prof. Lars L. Andersen, [lla@nfa.dk](mailto:lla@nfa.dk).
